# Dissemination and implementation science resources, training, and scientific activities provided through CTSA programs nationally: Opportunities to advance D&I research and training capacity

**DOI:** 10.1017/cts.2022.377

**Published:** 2022-04-22

**Authors:** Rachel C. Shelton, Rowena J. Dolor, Jonathan Tobin, Ana Baumann, Catherine Rohweder, Sapana Patel, Laura-Mae Baldwin

**Affiliations:** 1 Columbia University Mailman School of Public Health and Columbia’s Irving Institute for Clinical and Translational Research, New York, NY, USA; 2 Division of General Internal Medicine, Department of Medicine, Duke Clinical and Translational Science Institute, Duke University, Durham, NC; 3 Clinical Directors Network (CDN) and The Rockefeller University Center for Clinical and Translational Science, New York, NY, USA; 4 Division of Public Health Sciences, Department of Surgery, Washington University in St. Louis, St. Louis, MO, USA; 5 UNC Center for Health Promotion and Disease Prevention, University of North Carolina at Chapel Hill, Chapel Hill, NC, USA; 6 New York State Psychiatric Institute and Columbia University Vagelos College of Physicians and Surgeons, New York, NY, USA; 7 Department of Family Medicine and the Institute of Translational Health Sciences, University of Washington, Seattle, WA, USA

**Keywords:** CTSA, implementation science, mentoring, training, translational research

## Abstract

**Introduction:**

Clinical and Translational Science Award (CTSA) Program hubs are well-positioned to advance dissemination and implementation (D&I) research and training capacity nationally, though little is known about what D&I research support and services CTSAs provide. To address this gap, the CTSA Dissemination, Implementation, and Knowledge Transfer Working Group conducted an environmental scan of CTSAs (2017–2018).

**Methods:**

Of 67 CTSA institutions, we contacted 43 that previously reported delivering D&I research services. D&I experts from these institutions were emailed a survey assessing D&I resources, services, training, and scientific projects. Responses were categorized and double-coded by study authors using a content analysis approach.

**Results:**

Thirty-five of the 43 D&I experts (81.4%) responded. Challenges to CTSAs in developing and supporting D&I science activities were related to inadequate D&I science workforce (45.7%) and lack of understanding of D&I science (25.7%). Services provided included consultation/mentoring programs (68%), pilot funding/grants (50%), and workshops/seminars/conferences (46%). Training and workforce development in D&I were frequently identified as future priorities. Recommendations included increase training to meet demand (68.6%), accessible D&I tools/resources (34.3%), greater visibility/awareness of D&I methods (34.3%), consultation services (22.9%), and expand D&I science workforce (22.9%).

**Conclusions:**

CTSAs have tremendous potential to support the advancement and impact of D&I science across the translational continuum. Despite the growing presence of D&I science in CTSAs, continued commitment and prioritization are needed from CTSA and institutional leadership to raise awareness of D&I science and its value, meet training demands, and develop necessary infrastructure for conducting D&I science.

## Introduction

Over the past 15 years, the field of dissemination and implementation (D&I) science has emerged to help address the well-documented gap between research (e.g., evidence-based programs, practices, guidelines, treatments) and practice (e.g., what is routinely delivered across real-world healthcare and public health settings) [1]. D&I science has the potential to accelerate the speed with which translation and population health benefits and impact occur [2]. Dissemination and implementation are distinct but related domains of inquiry. Dissemination research focuses on understanding the factors that lead to widespread use of evidence-based information by a target population [3]. Implementation research focuses on methods, processes, frameworks, and strategies to promote the uptake, use, and integration of research findings and other evidence-based practices into routine practice in specific clinical and community settings, with the goal of improving quality of care, clinical, and population health outcomes [3–5].

The field of D&I science is interdisciplinary and inter-professional in nature [6,7]. It recognizes team science and stakeholder engagement as critical to addressing the successful and equitable dissemination and implementation of effective practices across diverse and complex contexts and populations [8–10]. While still relatively new, there has been tremendous growth and interest in the field both by researchers and scientific organizations, demonstrated by a) growing numbers of D&I-specific journals and special issues [11], b) expanding funding opportunities for D&I research (e.g., National Institutes of Health (NIH), Patient-Centered Outcomes Research Institute (PCORI), and Agency for Healthcare Research and Quality (AHRQ)), c) well-attended D&I conferences and trainings [12], and d) the publication of D&I science textbooks [1,13,14].

For the field of D&I science to continue to advance and meet growing interest and demand, increased training and research capacity for D&I at academic/research institutions is needed [15–19]. While there are a growing number of training programs offered nationally (e.g., Training Institute for Dissemination and Implementation Research in Health (TIDIRH); Training Institute for Dissemination and Implementation Research in Cancer; Implementation Research Institute (IRI); Institute for Implementation Science Scholars) [20–23], these training programs are highly competitive and do not fully meet the demand for capacity building in this rapidly growing field [12,19,24]. In particular, there is a need for diverse types of trainings to meet the needs of different stakeholders, including graduate students/trainees, senior faculty, and practitioners [25–27]. However, given the breadth of topics and methodologies that D&I encompasses and the interdisciplinary nature of the field [15], the wide-reaching scope of D&I also presents challenges in finding a clear “academic home” or dedicated infrastructure for supporting D&I science and training within academic or research institutions.

The Clinical and Translational Science Awards (CTSAs), funded through the National Institutes of Health-National Center for Advancing Translational Science (NIH-NCATS), can play a leading role in advancing the field by housing the infrastructure to support and facilitate D&I research and training that crosses disciplinary and methodological boundaries [28]. CTSAs fund major translational research infrastructure in over sixty academic medical research centers across the USA in 30 states and the District of Columbia. These CTSA “hubs” have a history and mission of fostering collaboration between multidisciplinary investigators to (1) facilitate innovative translational research and training across all stages of the translational continuum (e.g., basic, clinical, and population sciences); (2) provide training to facilitate workforce development; and (3) develop, demonstrate, and disseminate effective research tools and solutions to overcome translational roadblocks (NIH, CTSA grant PAR-18–464) [29].

Relatively, little empirical research has been conducted to understand to what extent and through what mechanisms CTSAs are supporting and facilitating D&I science. Morrato *et al.* conducted interviews and surveys with leadership from 18 CTSAs to advance understanding of comparative effectiveness research (CER), an area closely aligned with D&I research [29]. The authors found interest in using the CTSA infrastructure to accelerate the translation of CER evidence, and half of the sites (n = 9) reported what they perceived to be “moderate” activity in this area. However, respondents felt that CER translation was not prioritized and efforts were fragmented. In particular, designing teams that represent the full spectrum of translational research, including both scientists in earlier stages along the translational pipeline and implementation science researchers, was a challenge for CTSAs. Furthermore, numerous barriers to the D&I of CER evidence by CTSAs were identified, including lack of institutional awareness, well-established D&I methods, and clarity about the quality and utility of CER evidence. Additionally, limited number of faculty with appropriate expertise and funding support were cited [29].

NCATS has recognized the importance of D&I science to advancing translational research (NCATS FOA) [30]. There is a growing consideration of the synergies between translational research and D&I [28] and opportunities to leverage CTSA infrastructure to advance D&I research and practice [11,31]. In 2016, the CTSA Collaboration and Engagement Domain Task Force created a Dissemination, Implementation, and Knowledge Transfer (DI&KT) Workgroup to examine the potential role of D&I sciences across the translational research continuum [28]. While common approaches in D&I research are well-aligned with the mission of CTSAs (e.g., interdisciplinary team science approaches, community/clinical partnerships, community engagement) [32–34], little is known about what D&I research support and services are currently provided by CTSAs nationally.

An initial prior survey led by the DI&KT Workgroup was conducted with CTSA leaders (Principal Investigators and Administrators) to identify D&I science-related activities, barriers, and needed supports. This survey (with responses from 37 CTSAs) found that common barriers to conducting D&I science included funding, limited D&I science faculty, and lack of understanding of D&I science. Training and coordination of D&I activities across CTSAs were identified as useful supports for facilitating D&I research [35]. We report here on a follow-up survey conducted among those identified by CTSA site leadership (e.g., site Principal Investigators and Administrators) as “D&I experts” within CTSAs nationally, in order to provide a more in-depth environmental scan and assessment of D&I resources, infrastructure, services, training, and scientific projects provided through existing CTSA hubs.

## Methods

### Data Collection

An environmental scan of D&I science resources and services, training, and research projects either directly funded or indirectly supported by CTSA programs was conducted in 2017-2018 by the national CTSA program’s DI&KT Workgroup. The DI&KT Workgroup created a survey comprising open-ended and closed-ended questions asking for detail from D&I experts on existing CTSA D&I scientific activities in three domains: 1) D&I research program/resource, 2) D&I research training/workforce development, and 3) D&I scientific research projects. Similar to the survey of CTSA leaders [35], the first part of the environmental scan asked a diverse set of questions (see Supplementary Table 1).

### Survey

Survey questions administered to the D&I experts asked them to give detailed answers for each specific D&I science domain (program/resource, training/workforce development, and scientific research projects) at their CTSA institution as shown in Table 1. The survey defined “dissemination” and “implementation” based on NIH definitions [4] and provided examples of what did and did not count as D&I scientific activities (e.g., having a community engagement core, distributing a newsletter about CTSA activities did not count). D&I science activities were defined as “resources, programs, training opportunities, and scientific projects (related to D&I science) that are supported by your CTSA or involve collaborations with other groups conducting D&I science activities within your institution,” with a range of examples provided (e.g., consultation service for D&I research program/resource; training workshop for D&I workforce development; pilot funding for D&I scientific research projects).

**Table 1.** Specific items asked on the D&I environmental scan survey for D&I resources/programs, trainings, and scientific activities offered at each CTSA

Specific items asked
• Funded by CTSA, or not funded • Title and Description • If not CTSA-funded, then how does CTSA support or collaborate with this program/training/scientific activity • Type of faculty/staff funded and not funded by CTSA (MD, PhD, MS) • Who uses program/training/scientific activity • How many use program/training/scientific activity annually • Integration of program/training/scientific activity with other programs, modules, and activities • Promotion of program/training/scientific activity by CTSA within institution, and/or across CTSA consortium • Metrics used to measure success of program/training/scientific activity • Level of funding or other resources provided to program/training/scientific activity

CTSA, Clinical and Translational Science Award; D&I, dissemination and implementation.

Additionally, respondents reported whether their CTSA directly funded or indirectly supported D&I science activities under each domain (see Table 2). Respondents could name up to three directly funded and three indirectly supported D&I science activities under each domain (up to 6 activities for each domain; and up to 18 activities across all three domains). “Direct funding” was defined for respondents as CTSA-allocated funds (partial or full) for D&I science activities, while “Indirect support” was defined as promoting and/or collaborating on D&I science activities within their institution that are supported but not directly funded by the CTSA award. Respondents were also asked about challenges and barriers to providing such D&I science activities through CTSAs and recommendations for how to support and strengthen the D&I infrastructure within their site-specific CTSAs and across the larger CTSA Consortium (national network of CTSA hubs).

**Table 2.** Proportion of respondent CTSAs directly and/or indirectly supporting D&I Activities

	D&I Research Program/Resource^a^ (n = 35)	D&I Research Training/Workforce Development^b^ (n = 35)	D&I Scientific Research Projects^c^ (n = 35)
Direct CTSA funding (CTSA-allocated funds [partial or full] for D&I science activity)	21	20	21
	60.0%	57.1%	60.0%
Indirect CTSA support (D&I science activity within institution but not funded by CTSA award)	22	25	13
	62.9%	71.4%	37.1%
Either type of CTSA support	27	26	24
	77.1%	74.3%	68.6%

CTSA, Clinical and Translational Science Award; D&I, dissemination and implementation.

a *D&I science program/resource example:* D&I Research Core, consultation services.

b *D&I science training/workforce development example:* Training course or workshop on implementation science.

c *D&I scientific research project example:* Pilot funding for D&I research project, development of methods/measures for implementation research.

The last two survey questions assessed the priorities and needs of their CTSA sites, specifically: 1) “what are the goals for D&I science activities within your CTSA?” and 2) “what are the priority topics for D&I science activities within your CTSA?” The survey was reviewed and revised by the CTSA Collaboration and Engagement Domain Task Force Lead Team prior to data collection.

### Recruitment and Sampling

The sample for this survey included 37 D&I experts identified from the prior survey of CTSA Principal Investigators (PIs) and Administrative Directors, in which they were asked to identify D&I experts in their programs [35]. The current survey added 6 additional DI&KT Working Group members from CTSAs that did not respond to the Phase 1 survey (see Supplementary Fig. 1). The survey was programmed into REDCap (online data collection platform) [36] and sent by email to the sample of 43 D&I experts. Thirty-five of these 43 D&I experts (81.4%) responded to the email survey and 28 of those reported a D&I scientific activity (e.g., D&I research program/resource; D&I training/workforce development; D&I scientific research project) (see analysis below). Surveys were collected between June 6 and August 18, 2017, and exported into Microsoft Excel for analysis.

### Data Analysis

Data collected from open-ended responses to survey questions were reviewed, categorized, and double-coded by two study authors (RCS, LMB) using a content analysis approach [37,38]. Discrepancies were reviewed, and consensus was used to determine the final coding and categorization presented in the results and tables (see Table 3 for the full list of descriptive categories and codes (e.g., domains and sub-domains). For closed-ended questions (yes/no questions), we calculated proportions.

**Table 3.** Summary of D&I resources, training, and scientific activities reported by D&I experts, organized by key domains and sub-domains (collected among 28 of the 35 respondent CTSAs that named a D&I resource, training, or scientific activity)

Type of D&I activity	CTSA-funded, n (%)^a^ (N = 25 CTSAs)	Indirect CTSA support, n (%)^a^ (N = 14 CTSAs)	Total, n (%) (N = 28 CTSAs)^a^
*D&I Research Resource or Program*			
Consultation/mentoring	17 (68%)	5 (36%)	19 (68%)
Toolkits or shared resources	4 (16%)	1 (7%)	5 (18%)
D&I interest group or work group	4 (16%)	2 (14%)	6 (21%)
*D&I Training/Workforce Development*			
Workshops, seminar series, conferences	11 (44%)	7 (50%)	13 (46%)
D&I-specific training program	2 (8%)	2 (14%)	4 (14%)
D&I integrated as part of curriculum for training (KL2, CDA)	4 (16%)	2 (14%)	5 (18%)
Stand-alone D&I curriculum or course	4 (16%)	3 (21%)	5 (18%)
*D&I Scientific Research Projects*			
D&I pilot/methods grant funding	14 (56%)	0	14 (50%)
D&I research support/coordinator staffing	1 (4%)	0	1 (4%)

CDA, Career Development Award; D&I, dissemination and implementation; KL2, a type of career development award.

a Column percentages add to more than 100% since some Resources/Programs offer more than 1 activity.

## Results

### CTSA Site Characteristics

A total of 35 of the 43 surveyed CTSA sites participated (81.4%). CTSA sites in the respondent sample were similar across the 4 census regions: West (25.7%), Mid-West (22.9%), South (25.7%), and Northeast (25.7%).

### Proportion of CTSA Sites Directly and/or Indirectly Supporting D&I Science Activities

Among the 35 sites, over half reported using direct CTSA funding (e.g., partial or full CTSA-allocated funds) to support: (1) D&I research programs/resources (e.g., a D&I Research Core or consultation services; 60%); (2) D&I scientific research projects (e.g., pilot funding for D&I research projects, development of measures; 60%); and (3) D&I research training or workforce development (57%). A high proportion of sites reported indirect CTSA support for D&I science activities within their institution (e.g., not funded by the CTSA award) to facilitate training/workforce development (71.4%) and D&I research programs/resources (62.9%); in contrast, fewer (37.1%) reported using indirect CTSA support for D&I scientific research projects (see Table 2 for full details).

### Overview of Key D&I Scientific Activities Across CTSA Sites

Of the 35 responding CTSAs, 28 described at least one D&I scientific activity they conducted (e.g., D&I research resource/program, D&I training, or D&I scientific research project) (see Table 3 for full details). In the *D&I research resource/program* domain, CTSAs commonly reported providing: consultation/mentoring programs (68%), D&I Interest or work groups (21%), and toolkits they developed (or facilitate access to) to support D&I research (18%). With respect to the D&I *training and workforce development domain*, 46% of CTSAs reported having D&I workshops/seminars, 18% reported providing a stand-alone D&I curriculum/educational course, 18% reported integrating D&I training into an existing training curriculum (e.g., for KL2, career development awards), and 14% developed a D&I-specific training program (e.g., Mentored Training for Dissemination & Implementation Research in Cancer (MTDIRC)). For both D&I research resources/programs and training/workforce development, activities were commonly supported by both CTSA-funded and indirect CTSA support. For the *D&I scientific research project domain,* 50% of respondents (n = 14) reported having D&I pilot grant funding. Table 4 and Supplementary Table 5 provide both examples and a full list of CTSA-supported D&I scientific activities (domains and sub-domains) at 28 CTSA sites.

**Table 4.** *Examples of D&I scientific activities (domains and sub-domains) across CTSA institutions as of 2017/2018* ^a^

Type of Resource (Sub-Domain)	CTSA Support (Direct Funding or Indirect Support)	Name of Resource (CTSA)	Description & Metrics
***Domain: D&I Research Resource or Program***			
Consultation/Mentoring	Direct funding	Center for Community-Engaged Translational Research (UTHSC-Houston)	Consultations for D&I research for grant proposals and funded projects. *Example Metrics: Number of D&I consultations; number of funded studies following consultations*
Consultation/Mentoring	Indirect support	Consultation and workforce development for community clinicians engaged in Community-Engaged Research (CEnR) (Rockefeller)	Monthly meetings involving review of project progress, challenges, problem-solving, interim data analysis, data quality review, dissemination planning, and topical case or literature review, periodic webcasts. *Example Metrics: Project completion, collaborator retention, external funding, publication*
D&I Interest/Support Group	Direct funding	D&I Research Methods Working Interest Group, DIMwits (Mayo)	Monthly meetings, journal club-like group that supports institutional capacity building and a sort of academic home for aspiring D&I researchers. *Example Metrics: Number of members, frequency of meetings*
D&I Interest/Support Group	Direct funding	Implementation Science Workgroup (Kansas)	Monthly convening of investigators working in D&I science. *Example Metrics: Participation; whether sessions support competitive grant applications and encourage team science collaborations.*
D&I Interest/Support Group	Indirect support	Network Expertise in Implementation Science (Univ of Washington)	Network of academic investigators, students, and staff working in the area of D&I science *Example Metrics: Number of presentations, number of collaborative projects*
Toolkit/Shared Resource	Direct funding	Shared Decision-Making Implementation (Mayo)	Support for funding evaluation of implementation process and toolkit development to support scale and spread of shared decision-making interventions. *Example Metrics: Publications*
Toolkit/Shared Resource	Direct Funding	D&I Research Toolkits (WUSTL)	A set of nine toolkits that compile resources on key D&I research topics. *Example Metrics: “hits” or counts of use on website*
***Domain: D&I Training/Workforce Development***			
D&I Curriculum/Course	Direct Funding	I-Corps@CCTSI (Univ Colorado Denver)	Immersion training (customer discovery) to identify the value proposition and sustainable business model for CTSA innovation. (e.g., designing for dissemination and sustainability). *Example Metrics: Based on the NSF model; also use the Net Promoter Score*
D&I Curriculum/Course	Direct Funding	Annual D&I Short Course (Univ of Wisconsin)	Multi-day short course featuring National D&I experts and UW D&I faculty. Didactic and interactive format; including panels, roundtables, and small group grant reviews. *Example Metrics: Pre-and post-evaluation.*
D&I part of curriculum for training program	Direct Funding	UCSF-CTSI Training program in Implementation Science (UCSF)	Training – in-person and online. *Example Metrics:* *completion of certificate program, number of grants funded, publications*
D&I part of curriculum for training program	Direct Funding	KL2 – training track on D&I (Iowa)	Training track within KL2 on D&I. *Example Metrics:* *number of programs offered, number of scholars trained*
D&I-specific training program	Direct Funding	CTSA Translational Research Training Program (TL1) in T4 methods (e.g., translation to community/population health (UCLA)	Funding for predoctoral/postdoctoral fellows to develop T4 research methods. *Example Metrics: Total participants; some pre-post skills/knowledge assessment pertaining to specific coursework*
DI-specific training program	Funding	Implementation Research Institute and MTDIRC (WUSTL)	NIH grant-funded training programs in D&I research; nationally recruited fellows
Workshop/Conference/Seminar	Indirect Support	Implementation Science Training Series (Northwestern)	A series of workshops on implementation science and implementation research methods. *Example Metrics: Attendance at workshop; viewings of recordings.*
***Domain: D&I Scientific Research Activity***			
Funding for pilot project/methods	Direct Funding	Pilot grant program (NYU School of Medicine)	Pilot grants have been funded annually to support this type of research and help faculty obtain preliminary studies to support a larger grant. *Example Metrics: Subsequent grants & publications*
Funding for pilot project/methods	Direct Funding	CTSA/LA DHS Implementation Science RFA (UCLA)	Funding for implementation science research projects between CTSI investigators and the Los Angeles Department of Health Services (between $50K to $75K/year). *Example Metrics: Total participants; Amount of extramural funding; publications (academic and non-academic)*
Funding for pilot project/methods	Direct Funding	CTSA Pilot grants (UIC)	CTSA pilot grants fund projects that will advance translational science for up to two years. The pilot projects are expected to yield data that will enhance a federally funded grant. *Example Metrics: Milestones met; grants submitted and funded.*
Funding for pilot project/methods	Direct Funding	CTSI Translational Pilot Program (Wake Forest)	Open and targeted RFAs that are designed to “pull” pilot proposals from the community that address high-priority gaps and barriers. Implementation Science is one of the Program’s target areas. RFAs are released in the fall of each year and are for up to $40,000. *Example Metrics: Return on Investment of pilots including extramural funding, publications, abstracts, grant proposals*

CEnR, community-engaged research; CTSA, Clinical and Translational Science Award; CTSI, Clinical and Translational Science Institute; D&I, dissemination and implementation; DIMwits, Dissemination and Implementation Research Methods Working Interest Group; I-Corps@CCTSI, Innovation Corps Colorado Clinical and Translational Sciences Institute; KL2, a type of career development award; LA DHS, Los Angeles County Department of Health Services; MTDIRC, Mentored Training for Dissemination and Implementation Research in Cancer; NIH, National Institutes of Health; NSF, National Science Foundation; NYU, New York University; RFA, request for applications; UCLA, University of California Los Angeles; UCSF, University of California San Francisco; UIC, University of Illinois at Chicago; UTHSC, The University of Texas Health Science Center; UW, University of Wisconsin; WUSTL, Washington University in St. Louis.

a Please note, given that the data was collected in 2017–2018, it is possible that these CTSA programs have changed or been updated since then.

### Challenges/Barriers to Developing and Supporting D&I Science Activities

Among D&I experts (based on open-ended prompts), commonly reported challenges to CTSAs in supporting D&I science included inadequate D&I science workforce to meet growing demand (45.7%); lack of understanding and awareness of D&I science within academic institutions and CTSAs (25.7%); lack of funding to support D&I training and research (22.9%); and the need for buy-in and cultural shift within the scientific community and institutional leadership to understand the value of D&I science (22.9%) (e.g., prioritize and elevate not only bench and clinical science, but also the value of D&I science across the translational continuum). Other moderately common challenges to developing and supporting D&I research activities included lack of awareness of existing D&I science resources available within CTSAs and academic institutions (17.1%); challenges regarding where D&I “fits in” within existing CTSA structure (14.3%); and competing priorities of clinicians/staff to engage in D&I science (11.4%). Other barriers that were identified included lack of distinction between community engagement and D&I science within the CTSA (8.6%), lack of availability of D&I science mentors (8.6%), and lack of available D&I training for the full range of D&I learners (e.g., advanced and beginner) (8.6%) (see Supplementary Table 2 for full results).

### Facilitators to Support Researchers to include D&I Science Activities

Among D&I experts (based on open-ended prompts), commonly cited recommendations for facilitating D&I science across all phases of translational research within CTSAs included training (e.g., introductory/foundations of D&I for KL2 & TL1 trainees and faculty at all career stages, as well as more advanced topics on advancements in the field) (68.6%), tools and resources (e.g., shared curriculum, online training modules, examples of funded D&I grants, toolkits on D&I methods/foundations) (34.3%), greater visibility/awareness of D&I science methods and approaches (34.3%), consultation services (e.g., including lessons learned in how to develop/maintain consultation) (22.9%), expanded D&I science workforce (including administrators, clinicians, practitioners) (22.9%), and clear NCATS/NIH mandates and metrics for D&I (e.g., written into language of funding announcements) (20%). Other facilitators that were identified across multiple sites to support researchers in including D&I science activities included national coordination across CTSAs (e.g., through the Consortium) (17.1%), health system and community engagement (17.1%), allocation of CTSA resources (17.1%), and general collaboration (e.g., networking, exchanges of training resources/experts) across CTSA sites (14.3%). Facilitators mentioned less often included improved platform for dissemination (8.6%) and leveraging the CTSA’s parent institution’s resources (8.6%) (see Supplementary Table 3 for full results).

### CTSA Consortium Resources to Strategically Support D&I Science

Based on existing services/resources available to the larger CTSA, Consortium participants were asked to identify three services/resources that could be used more strategically to support D&I research (open-ended). Participating sites most frequently reported providing a common portal or refined compendium of key D&I tools/resources (e.g., curricula, courses, webinars, toolkits, example grants) (60%), facilitation of CTSA Consortium collaborations (57.1%), and shared educational materials/training (e.g., shared slide sets, modules) (42.9%) as CTSA Consortium resources that can be used strategically to support D&I science. Other services and resources that were reported, but to a lesser degree, included funding (e.g., pilot funds to facilitate cross-CTSA collaborations) (17.1%), centralized CTSA-based consultation services available to scientists (17.1%), collaborations across the CTSA to facilitate D&I science (e.g., matchmaking with national D&I experts, training exchanges) (14.3%), and building awareness of CTSA Consortium resources (11.4%) (see Supplementary Table 4).

### Future Priorities and Goals for D&I Science

Top goals and priorities identified by respondents for their specific CTSA sites (through open-ended prompts) were as follows: 1) D&I training/workforce development (51%) and 2) facilitating cross-institutional D&I research collaborations (37%). Respondents were more split across other goals and priorities, including building new D&I research infrastructure/initiatives (e.g., building consultation programs or work groups) (17.9%); tool/resource development (e.g., development of toolkits to facilitate use of D&I frameworks, strategies, methods) (17.9%); advancing the science of D&I (17.9%); and focusing D&I science on issues of health equity (17.9%). Less commonly reported goals and priorities were obtaining competitive D&I external funding (10.7%) and building greater awareness of D&I science at their institutions (7.1%). Additionally, 17.9% of respondents representing CTSAs said they had no future goals or they were not well defined.

## Discussion

CTSA goals and the objectives of D&I science are well-aligned and positioned to advance the translation of research to practice and ultimately improve clinical and population health [28]. CTSA hubs already provide infrastructure and “natural laboratories” for conducting team science, and D&I science can provide the theoretical frameworks, knowledge, and principles to address the research-to-practice gap. Despite the potential for a more explicit alignment of CTSAs with the field of D&I science, little empirical work has been conducted to examine the nature and extent to which CTSAs currently support and actively facilitate D&I research and training. This research, conducted with D&I experts from 35 CTSA sites nationally, was designed to address that gap and identify the barriers and supports to adopting and advancing D&I science within CTSAs.

Results suggest there is growing support of D&I science activities across many of the participating CTSAs from our sample: over 50% of the 35 participating CTSAs directly funded D&I science programs/resources (e.g., D&I Research Core, consultation service), and D&I scientific research projects (e.g., D&I pilot funding), and several funded D&I research training. Furthermore, when indirect CTSA support is added, an even higher proportion of CTSAs (68%-77%) reported CTSA support for these D&I scientific activities. However, despite the provision of some funding support for D&I activities within CTSAs, results suggest that D&I experts perceive a need for developing, expanding, and supporting the D&I workforce that could be facilitated by more formal integration and provision of infrastructure and directly funding resources to support and enhance the conduct of D&I science within CTSAs.

The most common type of CTSA-funded D&I science activity reported was consultation/mentoring for D&I research, followed by D&I pilot grant funding and D&I-focused workshops/seminars/conferences. While there is a growing D&I science presence within CTSAs, many programs were in a capacity building period and not yet formalized, and a number of key barriers were identified that impeded this development. The most commonly reported challenge to CTSAs developing and supporting D&I science was inadequate workforce (e.g., few faculty formally trained in D&I, and limited bandwidth for those who are trained given increasing demand and limited resources). Many of the other common barriers related to attitudes and norms, including lack of understanding of what D&I science entails at all levels of leadership and within the scientific workforce, and limited infrastructure within the existing CTSA structure. These barriers are largely consistent with those identified by Morrato *et al.* in examining deterrents to the D&I of CER evidence by CTSAs [29]. Insufficient capacity (e.g., mentors, resources) to meet expanding demand of D&I, lack of awareness of D&I as a field, and the broad scope and complexity of D&I science have been identified previously as some of the challenges to building institutional support for D&I science more generally, not specific to CTSAs [15,39]. Our findings are consistent with and expand upon the survey of national CTSA hub PIs by Dolor *et al.* which found similar challenges to conducting D&I science within CTSAs [35].

Recommendations for how CTSAs can better provide support for researchers to engage in D&I science and overcome these barriers included 1) the development and expansion of training and educational programs to meet the growing need for training the full range of D&I learners across all CTSA sites (e.g., K trainees, faculty at all career stages, practitioners); 2) the development of shared educational tools (e.g., open-access courses and curricula on introduction to D&I, D&I methods); 3) greater visibility and awareness of D&I and its value-added, as well as D&I methods for all researchers across the translational continuum; 4) sharing of lessons learned on delivering D&I consultation services; 5) more resources to support building a robust D&I science workforce across all hubs (e.g., training or recruiting experienced D&I researchers/mentors; covering faculty time for mentorship/training); and 6) clear NCATS/NIH mandates that prioritize a D&I focus. While D&I science training and growing the D&I science workforce and mentorship were also previously identified as key strategies to help develop and support D&I science activities within CTSAs [35], this survey provides further insight into potential facilitators and strategies for how CTSAs can actively support D&I science and importantly does so from the perspective of D&I experts at CTSA hubs.

These findings underscore the tremendous and ongoing need for training and education related to D&I for the wide range of potential D&I trainees. While there have been numerous workshops, institutes, courses, webinar series programs, and training programs over the past 12 years (e.g., TIDIRH, IRI, MTDIRC, National Heart Lung and Blood Institute K12) [12,19,20,39–41], the need and demand for training is greater than current capacity in reaching the specific needs of learners and diverse types of trainees across the translational continuum. Moreover, the currently available trainings tend to be disease-specific because of their funding mechanisms. CTSAs could be important hubs of a more generalized training capacity for D&I across different areas of expertise, giving an important platform to train and strengthen the field of D&I, increase the capacity of researchers to utilize D&I principles to move research into practice [25], as well as enhance team science approaches to bringing researchers and practitioners together. Additionally, training in community engagement approaches, including the healthcare delivery workforce, will be particularly important to advancing D&I science and ensuring its impact on population health outcomes.

Within CTSAs, it will be important to systematically address workforce development and institutional barriers for D&I workforce development and sustainment. Currently, the extent of integration of D&I into existing or new CTSA infrastructure or programming is at the discretion of individual CTSA sites. There are clearer expectations regarding engagement of D&I science in the recent funding opportunity announcement from NCATS [30] (e.g., each site is required to engage in D&I activities); however, greater prioritization of D&I science and expectations (e.g., clear metrics) by funders, and the needed resources to support this prioritization (e.g., in the Request for Applications), may help further address infrastructure-related barriers. The development of resources to accelerate and improve the effectiveness of consultations within CTSAs [24] was one recommendation identified by the participants of this study. Several CTSAs have developed self-service toolkits to facilitate the conduct and advancement of D&I research (e.g., toolkits for selecting implementation outcomes; toolkits facilitating selection of D&I theories, models, and frameworks) [42]. As suggested by respondents, other examples of educational resources could include repositories of D&I grants or funded research projects, best practices, case studies, and curricula. CTSAs (both as local sites and as a national Consortium) may play a central role in centralizing the wealth of resources available in the field of D&I (e.g., trainings, toolkits, websites) that can be overwhelming and difficult to navigate [43]. Having such common platforms will allow for cost savings in not having to replicate resources [29]. An “open-access,” centralized approach to D&I resources could be an effective approach to gather, index, store, and share D&I resources [44].

More explicit cross-CTSA collaboration could be beneficial in the form of mentoring and consultations to help build capacity at sites with fewer D&I resources and faculty. For example, more experienced sites could provide consultation and peer support as less experienced CTSAs further develop D&I educational programs. Another type of full-spectrum collaboration would model how to bring basic science and implementation science researchers together within a multi-CTSA project team [45]. Mentorship is recognized as an important component of many successful D&I national training programs [15,19,20,22,46], and, while this is already informally happening at institutions conducting D&I research, efforts to formalize mentorship through training programs or other modalities (e.g., matchmaking for D&I, peer-to-peer learning through “works in progress,” D&I networking opportunities or working groups, D&I consultation programs; videos; webinars; toolkits; cross-CTSA consultations) would be crucial to enhance and evaluate the building of D&I research capacity. Additionally, within existing CTSA training programs, CTSAs could have dedicated KL2, TL1 or career development slots focused specifically on D&I.

CTSAs can also leverage existing structures to integrate D&I training and research (e.g., community engagement core, workforce development, evaluation) [10]. There are excellent exemplars to learn from in building capacity for D&I research within CTSAs through community engagement [47]. For example, community engagement and practice-based research networks (PBRNs) [48] are central to D&I, and many CTSAs already have expertise in community engagement, PBRNs, and existing Community Advisory Boards, which D&I researchers could learn from as part of “best practices” in engaging with and learning from existing networks of patients, clinicians, or other community partners. This expertise and these networks could be better integrated to facilitate more bi-directional learning between D&I researchers and community partners/practitioners. Local stakeholders in community organizations can provide insight into the most pressing problems within communities and could leverage university resources to address those problems in collaboration with communities. Community partners can inform the feasibility and appropriateness of effective clinical or public health “innovations” or implementation strategies prior to D&I researchers’ efforts to implement them; when this is extended to include scientists working in earlier translational science phases (e.g., basic science), the research to practice to public health impact process may be accelerated.

To increase national visibility of D&I, CTSAs could provide clearer expectations and even mandates that promote D&I as a critical aspect of CTSA training and/or research, by allocating funds towards D&I, prioritizing pilot funds with D&I topics, and creating new D&I grant announcements that require cross-CTSA collaborations (alone, or in synergy with other agencies such as PCORI, AHRQ, Centers for Disease Control and Prevention, Health Resources and Services Administration, Veterans Affairs). In light of clinician/healthcare system competing priorities, incorporating team science and learning health systems approaches and partnerships with clinical healthcare delivery systems may be useful in synergizing quality healthcare delivery with implementation science, increasing both the generalizability and impact of this work. Additionally, PCORI [49] and CDC [50] are national organizations that have both adopted and applied D&I frameworks and principles to guide their research efforts (e.g., in informing partner engagement, evidence assessment, evaluation); aligned with such an approach, CTSAs may also benefit from adopting and refining D&I frameworks to further inform and advance their clinical and public health research efforts.

Our project’s limitations should be acknowledged. While our response rate was strong, we represented 35 CTSAs nationally, suggesting that we did not capture the full range of CTSA experiences. However, it is possible that CTSAs that do not have D&I programs or are not familiar with D&I did not reply to our survey; given that the CTSAs included here represent just over half of all CTSAs, it is likely that the gaps identified for building research and training capacity for D&I through CTSAs would be even greater. Further, given reports of low awareness of what D&I science entails, it is possible that we have underreported D&I science activities across CTSAs. Additionally, we captured the experience of CTSAs at one point of time and from the perspective of one individual representing their organization, which may also result in underreporting of D&I activities. However, the project’s strengths include (1) to our knowledge, the largest in-depth assessment of D&I resources, training, and programs at CTSAs from the perspective of local D&I science experts; (2) strong representation of CTSA sites regionally and geographically across the country; and (3) multiple investigators coded and categorized the data to enhance the reliability and validity of the open-ended response data (e.g., member checking between interviewers to discuss and resolve discrepancies).

In conclusion, the potential return on investment in D&I research and training is substantial, both in terms of building the infrastructure to create and sustain learning healthcare systems with the resulting impact on population health and health equity [51], as well as the benefits for researchers and trainees [19]. As identified through our findings and further elaborated upon here, CTSAs have the potential to lead nationally and provide critical resources, institutional commitment, infrastructure, and training to continue to advance the field of D&I science and expand its reach to investigators across the translational continuum, as well as to other local stakeholders (e.g., community members, patients, healthcare systems, providers). We encourage national and local CTSA leadership to more explicitly consider and formally adopt and test some of the opportunities and strategies suggested here for building greater D&I research and training capacity both within their local CTSAs and nationally as part of collaborative endeavors across the CTSA Consortium [52].

## Supplementary material

For supplementary material accompanying this paper visit https://doi.org/10.1017/cts.2022.377. click here to view supplementary material
