# Dissemination and implementation science resources, training, and scientific activities provided through CTSA programs nationally: Opportunities to advance D&I research and training capacity – ADDENDUM

**DOI:** 10.1017/cts.2022.406

**Published:** 2022-05-30

**Authors:** Rachel C. Shelton, Rowena J. Dolor, Jonathan N. Tobin, Ana Baumann, Catherine Rohweder, Sapana Patel, Laura-Mae Baldwin

**Keywords:** CTSA, implementation science, mentoring, training, translational research, addendum

The above article [1] published without Jonathan N. Tobin’s middle initial and without his ORCID ID.

The article has been updated online to include this missing information.
