# Supplementary material for: Dissemination and implementation science resources, training, and scientific activities provided through CTSA programs nationally: Opportunities to advance D&I research and training capacity
Source: J Clin Transl Sci. 2022 Apr 22;6(1):e41. doi: 10.1017/cts.2022.377 (PMC9066314; doi:10.1017/cts.2022.377)
Supplement: Supplementary file 1 [file S2059866122003776sup001.docx]

**Supplementary Material**

**Supplementary Table 1.** *Domains and Specific Items Asked on the Dissemination and Implementation Environmental Scan Survey*

| **Domain** | **Specific items asked** |
| --- | --- |
| D&I general questions (repeated from Dolor et al, 2019 paper)^35^ | - Whether the CTSA directly funds: 1) D&I science programs/resources (e.g. D&I consultation services), 2) D&I science trainings/workforce development (e.g. D&I workshop), or 3) D&I scientific research projects (e.g. D&I pilot funding); (note: direct support was defined as CTSA-allocated funds, partial or full, for D&I science activities); - Whether the CTSA collaborates on or supports (without use of CTSA funds, defined as indirect support) the same three D&I science activities listed above; - If D&I science activities are funded, the challenges or barriers they encountered in developing and supporting D&I science activities within their CTSA; - If no D&I science activities are funded by the CTSA, the reasons why; - To list up to three things that would help their CTSA program in supporting researchers to include D&I science activities across all phases of translational research (besides funding); - To identify three existing services/resources available to the larger CTSA Consortium that can be used more strategically to support D&I science within the overall CTSA program. |
| D&I Resource/Program | - Funded by CTSA, or not funded - Title and description - If not CTSA funded, then how does CTSA support or collaborate with this program - Type of faculty/staff funded and not funded by CTSA (MD, PhD, MS) - Who uses program - How many use program annually - Integration of program with other programs, modules, and activities - Promotion of program by CTSA within institution, and/or across CTSA consortium - Metrics used to measure success of program - Level of funding or other resources provided to D&I program |
| D&I Training/Workforce Development | - Funded by CTSA, or not funded - Title and description - If not CTSA funded, then how does CTSA support or collaborate on the training activity - Type of faculty/staff funded and not funded by CTSA (MD, PhD, MS) - Who uses training - How many are trained - Integration of training with other programs, modules, and activities - Promotion of training by CTSA within institution, and/or across CTSA consortium - Metrics used to measure success of training - Level of funding or other resources provided to D&I program |
| D&I Scientific Activity | - Funded by CTSA, or not funded - Title and description - If not CTSA funded, then how does CTSA support or collaborate on the scientific activity - Type of faculty/staff funded and not funded by CTSA (MD, PhD, MS) - Integration of scientific work with other programs, modules, and activities - Communication of scientific work by CTSA across CTSA consortium - Metrics used to measure success of scientific activity - Level of funding or other resources provided to D&I scientific activity |
| Priorities & Needs | - What are the goals for D&I science activities within your CTSA - What are the priority topics for D&I science activities within your CTSA |

CTSA, Clinical and Translational Science Award; D&I, dissemination and implementation.

**Supplementary Table 2.** *Challenges/Barriers to Developing/Supporting D&I Science Activities at CTSAs*

| Challenges/barriers | CTSAs reporting (n=35)  N (%) |
| --- | --- |
| - *Inadequate D&I science workforce* | 16 (45.7%) |
| - *Lack of understanding of D&I science* | 9 (25.7%) |
| - *Funding* - *Cultural shift* | 8 (22.9%) |
| - *Lack of awareness of D&I science resources* | 6 (17.1%) |
| - *Limitation of existing CTSA structure* | 5 (14.3%) |
| - *Engagement and competing priorities of clinician workforce/health systems* | 4 (11.4%) |
| - *Lack of distinction between CE and D&I* - *Lack of D&I science mentors* - *Training* | 3 (8.6%) |
| - *Inexperience with D&I science* - *Lesser priority in the national CTSA program* | 2 (5.7%) |
| - *Inadequate IT resources to support D&I science* - *High demand from investigators* - *Lack of interest* - *Building new connections among institutions’ D&I investigators* | 1 (2.9%) |

Respondents provided responses, unrestricted in number and length, to the following open-ended question: ‘What are some of the challenges or barriers you’ve encountered in developing and supporting D&I research activities within your CTSA?’. CE, community engagement; CTSA, Clinical and Translational Science Award; D&I, dissemination and implementation; IT, information technology.

**Supplementary Table 3.** *How CTSAs Can Support Researchers to Include D&I Science Activities across all Phases of Research (excludes funding)*

| Support | CTSAs reporting (n=35)  N (%) |
| --- | --- |
| - *Training* | 24 (68.6%) |
| - *Tools and resources* - *Greater visibility/awareness of D&I science methods* | 12 (34.3%) |
| - *Consultation service* - *More D&I science workforce* | 8 (22.9%) |
| - *Clear NCATS/NIH mandates* | 7 (20.0%) |
| - *National Coordination* - *Health system and community engagement* - *Allocation of CTSA resources* | 6 (17.1%) |
| - *General collaboration* | 5 (14.3%) |
| - *Better dissemination platforms* - *Leverage parent institution's resources* | 3 (8.6%) |
| - *Training peer reviewers* - *D&I science core in the CTSA* - *Local CTSA leadership support* | 1 (2.9%) |

Respondents were able to provide up to three responses, unrestricted in length, to the following open-ended question: ‘Not including funding, name up to three things that would help your CTSA Program support researchers to include dissemination and implementation research activities across all phases of research (e.g. T0 Basic Science through T4 Population Health Research).’ CTSA, Clinical and Translational Science Award; D&I, dissemination and implementation; NCATS, National Center for Advancing Translational Sciences; NIH, National Institutes of Health.

**Supplementary Table 4.** *CTSA Consortium Services and Resources that can be Used to Strategically Support D&I Science*

| Services and resources | CTSAs reporting (n=35)  N (%) |
| --- | --- |
| - *Tools/resources* | 21 (60.0%) |
| - *Collaboration across CTSA Consortium* | 20 (57.1%) |
| - *Educational materials/training* | 15 (42.9%) |
| - *Funding* - *Consult services* | 6 (17.1%) |
| - *Other/Collaborations outside CTSA* | 5 (14.3%) |
| - *Lack of awareness of CTSA Consortium resources* - *Informatics, especially related to health care systems* | 4 (11.4%) |
| - *Pilots* | 3 (8.6%) |
| - *CTSA Community Engagement module* - *CTSA Training programs (e.g., KL2, TWD)* - *Common metrics* - *Marketing and communications* | 2 (5.7%) |
| - *Biostatistics, Epidemiology and Research Design* - *D&I science theme groups* - *Multi-hub studies* - *Integrating Special Populations module* - *Trial Innovation Network* | 1 (2.9%) |

CTSA, Clinical and Translational Science Award; D&I, dissemination and implementation; KL2, a type of career development award; TWD, Translational Workforce Development.

**Supplementary Table 5.** *Full List of CTSA-Supported D&I Activities Across Resources, Training and Scientific Activities at 28 CTSA Sites Nationally*^a^

| **Type of Resource** | **CTSA Support (Direct or Indirect Funding)** | **Name of Resource (CTSA)** | **Description** |
| --- | --- | --- | --- |
| ***D&I Research Resource or Program*** | | | |
| Consultation/Mentoring | Direct | Population Health Research Consulting (Harvard) | Offers a cadre of research and evaluation consultants to provide teams of Harvard faculty and students, researchers from other universities, and community partners with methodological assistance in community-engaged research. This includes training and consultation on mixed methods, and implementation and dissemination science, policy, and community-based participatory research. |
| Consultation/Mentoring | Direct | Consultation Service (NYU) | Provides consults to faculty writing grants that require a D&I component. |
| Consultation/Mentoring | Direct | Consultation/Mentoring (UCSD) | Provide mentoring on career development awards (formal) and ad-hoc consultation on D&I issues for grants and manuscripts to post-baccalaureates, graduate students, post-docs, and junior faculty. |
| Consultation/Mentoring | Direct | D&I Navigator (Kansas) | This individual helps navigate investigators to relevant D&I expertise (new resource to be implemented soon after survey completion). |
| Consultation/Mentoring | Direct | Consultation on design of D&I research projects (USC) | Consultative services for clinician investigators interested in conducting D&I research in the LA County Safety-Net system. |
| Consultation/Mentoring | Direct | Center for Community-Engaged Translational Research (UTHSC-Houston) | Consultations for D&I research for grant proposals and funded projects. Consultations available to both junior and senior faculty and all CTSA investigators. |
| Consultation/Mentoring | Direct | Dissemination and Implementation Consultation Service (Univ of Washington) | One-on-one consultation between academic investigators and D&I Science experts to support grant development and project execution in D&I science. |
| Consultation/Mentoring | Direct | D&I Research Consultation (Univ Wisconsin) | Short-term and ongoing consultation services, such as D&I study design, frameworks, funding opportunities, dissemination plans, mentoring for early stage D&I researchers, assisting with mixed methods evaluation plan for D&I research. Services available to UW and Clinic Faculty, Trainees and staff. |
| Consultation/Mentoring | Direct | The Launchpad (Univ of Wisconsin) | New Program and Infrastructure to: Identify interventions that are ready for dissemination, have high demand, high potential for impact, high potential for financial sustainability, and lack commercial partners. Work with 1-2 investigators per year to provide project management, market research, business plan, legal assistance with intellectual property assistance, packaging, training & technical assistance. Begin product sales and engage in iterative pilot tests in preparation for wider spread. Identify home for sustainable product sales. Program available to researchers who develop interventions. |
| Consultation/Mentoring | Direct | The Learning Health System (Vanderbilt) | The Learning Health System (LHS) is a platform that can be utilized by researchers at VUMC to conduct randomized, clinical trials in a pragmatic fashion. The platform was funded as an optional module in the most recent CTSA renewal. It engages with the healthcare system to perform effectiveness trials, evaluating outcomes and process metrics relevant to stakeholders. Investigators who wish to implement interventions in the LHS submit an application to the LHS review board which determines which projects align with the mission of the LHS and are feasible in the LHS platform. |
| Consultation/Mentoring | Direct | D&I Research Bootcamp (Wash Univ St. Louis) | One-day intensive consultation for D&I research grants. Available to Institute of Clinical and Translational Sciences faculty preparing grants in D&I research. |
| Consultation/Mentoring | Indirect | Consultation and Workforce development for community clinicians engaged in CEnR (Rockefeller) | Monthly in-person meetings or teleconferences involving review of project progress, challenges, problem solving, interim data analysis, data quality review, dissemination planning, and topical case or literature review. Also periodic webcasts to a national audience. Participants include community clinicians, patient and other community stakeholders, research team members, laboratory investigators, Clinical Scholars and postdocs. |
| Consultation/Mentoring | Indirect | UAMS Center for Implementation Research (Univ of Arkansas) | Center funded by institution to build capacity in D&I. Very aligned with CTSA goal, but a separate institution. |
| Consultation/Mentoring | Indirect | Research to Advance Community Health (ReACH) Center (UTHSC - San Antonio) | An interdisciplinary research center for health services, D&I, and community-based research for research investigators. |
| D&I Interest/Support Group | Direct | D&I Research Methods Working Interest Group, DIMwits (Mayo) | Monthly meetings, journal-club like group that supports institutional capacity building and a sort of academic home for aspiring D&I researchers. Participants include junior health services researchers and other investigators and clinicians aiming to increase D&I knowledge and ability. |
| D&I Interest/Support Group | Direct | Implementation Science Workgroup (Kansas) | Monthly convening of investigators working in D&I science. This is an open workgroup, so all interested investigators are welcome to attend. |
| D&I Interest/Support Group | Direct | Implementation Science Affinity Group (Wake Forest) | The mission is to engage and support investigators in the area of dissemination and implementation research. Our goal is to translate evidence-based, clinical and health promotion strategies into real-world settings. Available to faculty, students/trainees, and staff. |
| D&I Interest/Support Group | Indirect | Network Expertise in Implementation Science (Univ of Washington) | Network of academic investigators, students and staff working in the area of D&I science. |
| Toolkit/Shared Resource | Direct | Shared Decision Making Implementation (Mayo) | Support for funding evaluation of implementation process and toolkit development to support scale and spread of shared decision making interventions. |
| Toolkit/Shared Resource | Direct | D&I Research Toolkits (WUSTL) | Set of nine toolkits that compile resources on key D&I research topics. Available to investigators affiliated with our CTSA; investigators nationally. |
| ***D&I Training/Workforce Development*** | | | |
| D&I Curriculum/Course | Direct | D&I Online Course (Harvard) | https://catalyst.harvard.edu/courses/implementation-research |
| D&I Curriculum/Course | Direct | I-Corps@CCTSI (Univ Colorado Denver) | Immersion training (customer discovery) to identify the value proposition and sustainable business model for a clinical and translational sciences innovation. This fits within the portfolio of designing for dissemination and sustainability. Participants include the full range of T1-T4 researchers: faculty, students, and clinical staff. |
| D&I Curriculum/Course | Direct | Annual D&I Short Course (Univ of Wisconsin) | Multi-day short course featuring national D&I experts and UW D&I faculty. Didactic and interactive format; including panels, roundtables, and small group grant reviews. Participants include faculty, academic staff, trainees, and community partners. |
| D&I Curriculum/Course | Indirect | D&I Masters level courses (Harvard) | 2 D&I courses recently launched at Harvard School of Public Health - one in Health Policy and Management and one is Social and Behavioral Science. Participants include Masters and Doctoral students at Harvard School of Public Health. |
| D&I Curriculum/Course | Indirect | Implementation Science Course (NYU) | Launched one-week course as part of CE certificate program and also includes scholars from CTSI masters program. Participants include faculty and post docs across NYU. |
| D&I Curriculum/Course | Indirect | Translational Science Research Fellowship (UTHSC - San Antonio) | D&I training embedded in research course for fellows and doctoral students. Available to trainees and junior faculty seeking D&I education within the CTSA. |
| D&I part of curriculum for training program | Direct | Epidemiology and research design tutorials (Rockefeller) | Case studies of specific research methods including D&I are presented by Clinical Scholars; hypothetical proposed studies are developed and discussed to illustrate the principles of study design, including D&I research. Tutorial attendance is a required part of Clinical Scholars training; faculty, post doctoral students and staff are invited to attend according to their interests. |
| D&I part of curriculum for training program | Direct | UCSF-CTSI Training program in Implementation Science (UCSF) | Training - in person and on-line. Available to all faculty and fellows, a wide range of community members. |
| D&I part of curriculum for training program | Direct | Introduction to D&I Research graduate course (Univ Colorado Denver) | 2-credit course offered annually. Participants include graduate students and career development awardees. |
| D&I part of curriculum for training program | Direct | KL2 - training track on D&I (Iowa) | Training track within KL2 on D&I. Participants are KL2 scholars. |
| D&I part of curriculum for training program | Direct | Master of Science in Clinical Investigation (UTHSC - San Antonio) | Master of Science in Clinical Investigation. Participants include clinical fellows, junior faculty. |
| D&I part of curriculum for training program | Direct | KL2 Scholars (UTHSC - San Antonio) | Mentored research award. Participants include junior faculty with institutional appointment. |
| D&I part of curriculum for training program | Indirect | AHRQ PRIME training program (Einstein) | AHRQ funded R25 education and training program in patient-centered outcomes research. |
| D&I part of curriculum for training program | Indirect | Performance Improvement fellowship (Einstein) | 12-month intensive training program for clinicians, healthcare providers, administrators, and associates at Montefiore. The goal of the fellowship program is to develop future PI leaders across the Montefiore. |
| D&I-specific training program | Direct | CTSA Translational Research Training Program (TL1) in T4 methods (e.g. translation to community/population health (UCLA) | Funding for predoctoral and postdoctoral fellows to develop capabilities in T4 research methods. |
| D&I-specific training program | Indirect | Implementation Research Institute and MTDIRC (WUSTL) | NIH grant-funded training programs in D&I research; nationally recruited fellows and junior faculty. |
| D&I-specific training program | Indirect | Massachusetts Consortium for Cardiopulmonary Implementation Science KL2 (U Mass) | NHLBI-funded KL2 program. Participants are junior faculty. |
| D&I-specific training program | Indirect | Prevention and Control of Cancer: Training in Implementation Science (PRACCTIS) | NCI-funded R25 post-doc program. |
| Workshop/Conference/Seminar | Direct | Late-Stage Translational Research Program (Mayo) | Optional module devoted to late-stage translational research and institutional capacity building and resource for D&I. Participants include institutional investigators across virtually all clinical and research departments for the purposes of consultation or grant partnership; also junior health services researchers desiring to gain D&I expertise. |
| Workshop/Conference/Seminar | Direct | Implementation Science Series (UC Irvine) | Co-sponsored with the School of Nursing. Bring D&I experts to give presentations, sit down for learning discussions, and have roundtables to work out D&I study ideas. Participants include faculty from nursing, medicine, social ecology, and other disciplines. |
| Workshop/Conference/Seminar | Direct | Implementation Science Seminar (UCSD) | Monthly seminar on D&I issues. Participants include early to senior career researchers. |
| Workshop/Conference/Seminar | Direct | Clinical Bootcamp (UIC) | Program for faculty and staff to learn more about clinical translational research. |
| Workshop/Conference/Seminar | Direct | Dissemination and Implementation Science 101 (Kentucky) | Training funded by CTSA pilots program and open to all interested. |
| Workshop/Conference/Seminar | Direct | Research to speed translation: D&I theories and methods (UTHSC-Houston) | Training on the basics of D&I, covering: what the field of D&I is (and is not), why it is important, what it is trying to achieve, how it is relevant to their projects, major components of a D&I study, D&I theories, models and frameworks, design considerations. Participants include graduate students, postdoctoral fellows, and faculty interested in implementation science. |
| Workshop/Conference/Seminar | Direct | Health Promotion Planning: An Intervention Mapping Approach (UTHSC-Houston) | Instruction on Intervention Mapping. Participants learn about the systematic approach to designing behavior change interventions; this includes adaptation of evidence-based interventions and implementation interventions. Participants include graduate students, postdoctoral fellows, faculty, community members. |
| Workshop/Conference/Seminar | Direct | Dissemination and Implementation Science Workshop (Univ of Washington) | Half-day workshop on the science of D&I, offered both in person at our CTSA and remotely to regional institutions. Participants include academic investigators and students interested in learning more about D&I science. |
| Workshop/Conference/Seminar | Direct | Individual Consultation on D&I Research Grants (WUSTL) | CTSA affiliated PIs receive individualized technical assistance on D&I research grants. |
| Workshop/Conference/Seminar | Indirect | Univ of Wisconsin D&I Short Course (Mayo) | Mayo supports travel for investigators to attend 2-day D&I Course at U of Wisconsin. Participants include junior PhD researchers/postdocs, clinician investigators. |
| Workshop/Conference/Seminar | Indirect | Implementation Science Training Series (Northwestern) | A series of workshops on implementation science and implementation research methods. Participants include faculty and trainees university wide. |
| Workshop/Conference/Seminar | Indirect | Southern California improvement and implementation science symposium (UCSD) | Host institutions include the Clinical and Translational Science Institutes at UCLA, USC, UC San Diego, UCI and UCR, the Gehr Family Center for Health Systems Science along with the Los Angeles County Health Agency, Kaiser Permanente Southern California, the Department of Veterans Affairs Greater Los Angeles Healthcare System, and RAND. |
| Workshop/Conference/Seminar | Indirect | ACCORDS Education (Univ Colorado Denver) | Annual education series of workshops and training. Some examples include: mixed methods; designing for dissemination; quality research methods forum. Past workshops included: introduction to D&I and introduction to pragmatic trials research. Participants include T3 and T4 researchers. |
| Workshop/Conference/Seminar | Indirect | Implementation Research Seminar Series (Kansas) | Seminar series supported in conjunction with the Cancer Center. Available to KL2 and TL1 trainees. |
| Workshop/Conference/Seminar | Indirect | Ad Hoc D&I Science Presentations (Univ of Washington) | Faculty deliver D&I science presentations to a variety of stakeholders, i.e. research seminars, clinical workgroups, research funders (NIH Kidney Health Initiative Stakeholders Meeting), presentations at ACTS meetings. |
| ***D&I Scientific Research Project*** | | | |
| Funding for pilot project/methods | Direct | Pilot grant program (NYU) | Pilot grants have been funded annually to support this type of research and help faculty obtain preliminary studies to support a larger grant. Pilot grants encourage collaboration across the university and with clinical partners. |
| Funding for pilot project/methods | Direct | D&I Pilot Project Grants (Northwestern) | Small grants ($10K) for D&I projects of Northwestern faculty. |
| Funding for pilot project/methods | Direct | D&I Pilot grant program (Univ of Arkansas) | CTSA just funded a round of pilot grants in D&I (4 recently funded). |
| Funding for pilot project/methods | Direct | CTSA/LA DHS Implementation Science RFA (UCLA) | Funding for several implementation science research projects undertaken between CTSI investigators and the Los Angeles Department of Health Services (between $50K to $75K/year). This includes 4 projects in 2015, 3 projects in 2016, and 2 projects in 2017. |
| Funding for pilot project/methods | Direct | D&I Pilot studies (UCSD) | Pilot studies that pair junior and more advanced investigators in engaging in D&I pilot studies. The CTRI funds roughly two D&I pilot studies per year. |
| Funding for pilot project/methods | Direct | Learning Health System Translational Pilot Program (Univ of Florida) | Provides pilot funds for implementation science studies that support the learning health system. Users include: Scientists, patients who are interested in presenting their ideas for research, clinicians with research topics. |
| Funding for pilot project/methods | Direct | CTSA Pilot grants (UIC) | CTSA pilot grants fund projects that will advance translational science for up to two years. The pilot projects are expected to yield data that will enhance a federally funded grant (open to all faculty). |
| Funding for pilot project/methods | Direct | CTSA Pilot grants (Iowa) | D&I projects are eligible for CTSA pilot grants. |
| Funding for pilot project/methods | Direct | D&I Research Award Program (Univ of Wisconsin) | This funding opportunity solicits grant applications that will identify, develop, test, evaluate and/or refine strategies to disseminate and implement evidence-based practices into public health, clinical practice, and community settings. Examples of evidence-based practices include behavioral interventions; prevention, early detection, diagnostic, treatment and disease management interventions; quality improvement programs. Award: Up to $150,000 for 18 months of direct cost support. Users include university investigators. |
| Funding for pilot project/methods | Direct | CTSI Translational Pilot Program (Wake Forest) | Releases both open and targeted RFAs that are designed to 'pull' pilot proposals from the Wake Forest Community that address high-priority gaps and barriers. Implementation Science is one of the Program's target areas. RFAs are released in the fall of each year and are for up to $40,000. These are one-year awards. |
| ***Multiple D&I Activities*** | | | |
| Consultation/Mentoring; Workshop/Conference/Seminar | Direct | Dissemination and Implementation Research and Policy Program (UIC) | Provides individual consultation to university researchers, both inside and outside the university, on integrating implementation and dissemination science into their research as well as designing D&I studies. We also partner with our education group to support D&I workforce development. Participants include researchers from our university and nationally. |
| Consultation/Mentoring; Funding for D&I projects/methods | Direct | Community System Engineering for Health Promotion Program Implementation (Mayo) | Support for a community partnership focused on the D&I of evidence-based health promotion programs and the study of this process. |
| Consultation/Mentoring; Toolkit/Shared Resource; D&I Interest/Support group; Workshop/Conference/Seminar; D&I-specific training program | Indirect | Adult and Child Consortium for Health Outcomes and Research Delivery Science, ACCORDS (Univ Colorado Denver) | Create collaborative learning partnerships with embedded research settings to translate research into practice more quickly and successfully. Conduct cutting edge translational research on: pragmatic research and measures, adaptation of interventions, designing for dissemination, shared decision making, planning for and evaluation of reach, implementation and sustainability. Use interactive on-line resources and support for patients, medical and public health students, trainees and faculty researchers. Communicate frequently updated information on D&I related conferences, articles, grant opportunities, events, webinars, talks, and training opportunities. Provide local consultation on D&I related research to increase funding and publication success. |
| Consultation/Mentoring; Toolkit/Shared Resource; Funding for D&I pilot project/methods | Direct | D&I Methods Unit (UNC) | The Methods Unit is part of the Community Engagement Core and is funded to provide consultations and resources to investigators. They engage in activities to further the science of D&I Methods through instrument development and applying for grant funds available to CTSAs to conduct Implementation Science. Participants include faculty and staff at UNC and other universities, collaborators from CBOs in North Carolina. |
| Consultation/Mentoring; Workshop/Conference/Seminar | Indirect | Improvement Science Research Methods (UCLA) | Offer academic clinicians within the CTSI's health system a series on improvement science, which is a 5-session series over 6 months that includes coaching on a project. Participants are academic clinicians. |
| Consultation/Mentoring; Workshop/Conference/Seminar; D&I Curriculum/Course | Direct | Implementation Science Module (Univ of Arkansas) | No description provided. Participants include researchers, fellows, residents, and students at UAMS. |
| Consultation/Mentoring; Workshop/Conference/Seminar; D&I Curriculum/Course | Direct | Pragmatic Trials and Dissemination/Implementation Research Unit (Univ Colorado Denver) | Seeks to: 1) foster a learning community and serve as a conduit for active dissemination of resources from NIH and other national organizations engaged in implementation science and pragmatic research. 2) To catalyze and centrally support the CCTSI community in the development of pragmatic trials, comparative effectiveness research methods, and implementation science to achieve increased efficiency and economies of scale. 3) Catalyze and centrally support the active bi-directional D&I of (a) CTSA-consortium innovation within the CCTSI and (b) select CCTSI innovation across the CTSA network in a systematic way to advance dissemination and translational science. Broad usage across our membership - School of Medicine, Colorado School of Public Health, School of Pharmacy, and College of Nursing - and at our partner institute (Colorado State University). Participation from junior and senior faculty; fellows & researchers. |
| Consultation/Mentoring; Workshop/Conference/Seminar;Funding for D&I pilot projects/methods | Direct | Dissemination, Implementation, Improvement Science Initiative (UCLA) | Promotes D&I methods through training; consultation to investigators as they develop protocols/proposals; matchmaking of investigators with community partners; promoting methods by offering communitywide symposia and webinar series within the CTSI; also (for 2 years so far) a collaborative RFA between UCLA and USC for proposals involving implementation science and the Los Angeles Department of Health Services. Participants include UCLA CTSI investigators; cross-CTSI investigators; community partners (health systems, CBOs, public agencies). |
| Consultation/Mentoring; Workshop/Conference/Seminar;Funding for D&I pilot projects/methods; Coordinator Support for D&I projects | Direct | The Collaboratory for Implementation Science (Dartmouth) | Support implementation research (IR) pilot studies conducted by clinician researchers by providing research design consultation, methodological support, and part-time research coordinators and/or research assistants for brief (6-12 month) IR pilot studies. In addition, we provide mentoring of clinician investigators in IR and a new seminar series to help grow a cadre of clinician implementation researchers. The focus of this program is on clinician researchers supported by experienced PhD and MD faculty and collaborators through team science. |
| D&I Interest/Support Group; Workshop/Conference/Seminar | Direct | Implementation Science Summit (UCSD) | Two-day summit across school of medicine to bring in internationally known D&I keynote, and other activities such as breakout sessions, workshop grant proposals, poster presentations all around D&I issues. Participants include any academic or research investigators at varying levels from grad students, medical students, residents, fellows, and faculty. |
| Toolkit/Shared Resource; Funding for D&I pilot project/methods | Direct | Researcher readiness for participating in community-engaged dissemination and implementation (CEDI) research (UNC) | We have created a conceptual framework that specifies competencies for researchers participating in community-engaged D&I research (CEDI) and maps these competencies to domains. Our next steps are to conduct further validation of the framework, then develop a CEDI research readiness assessment tool that measures a researcher's attitudes, willingness, and self-reported ability to effectively conduct community-engaged research. The tool, once developed, will guide participants to training resources to address identified needs. We are currently writing a U grant with two other CTSAs that will further the work of the CEDI and result in a survey instrument. Internally, the instrument will be used to identify areas of training for faculty. |
| Toolkit/Shared Resource; Funding for D&I pilot project/methods | Direct | Theory and Framework Selection Tool (UNC) | We will develop a user-friendly, disease-agnostic tool to guide model application for implementation scientists. The tool will include checklists of steps for applying models as well as examples of high-quality model application in manuscripts and grant applications. We will assess the tool using cognitive interviews with implementation researchers and community stakeholders. We will disseminate the tool via our online D&I portal and by integrating it into an existing web algorithm for model selection, as well as through a regional conference. |
| Toolkit/Shared Resource; Funding for D&I pilot project/methods | Direct | Classifying Implementation Strategies (UNC) | Faculty with the D&I Methods Unit developed an approach to categorize implementation strategies based on who enacted the strategy (i.e., the actor) and the level and determinants that were targeted (i.e., the action targets). We will continue this work and begin to integrate D&I theory. |
| Toolkit/Shared Resource; Funding for D&I pilot project/methods | Direct | Develop and Test Methods to Advance Dissemination and Implementation Science (Univ of Washington) | Identify gaps in D&I science, conduct scientific work to address these gaps, evaluate and disseminate results of D&I science. One completed project led to development of a tool that clinicians use to measure the implementability of an evidence-based intervention. Ongoing work will develop a tool that investigators can use to tailor implementation strategies to the baseline environment of health systems. |

ACCORDS, Adult & Child Consortium for Health Outcomes Research & Delivery Science; ACTS, Association for Clinical and Translational Science; AHRQ PRIME, Agency for Healthcare Research and Quality PRIME; CBO, community-based organization; CE, continuing education; CEDI, community-engaged dissemination and implementation; CEnR, community-engaged research; CTRI, Clinical and Translational Research Institute; CTSA, Clinical and Translational Science Award; CTSI, Clinical and Translational Science Institute; D&I, dissemination and implementation; DIMwits, Dissemination and Implementation Research Methods Working Interest Group; I-Corps@CCTSI, Innovation Corps Colorado Clinical and Translational Sciences Institute; IR, implementation research; KL2, a type of career development award; LA DHS, Los Angeles County Department of Health Services; LHS, The Learning Health System; MTDIRC, Mentored Training for Dissemination and Implementation Research in Cancer; NCI, National Cancer Institute; NHLBI, National Heart, Lung, and Blood Institute; NIH, National Institutes of Health; NYU, New York University; PI, performance improvement; PRACCTIS, Prevention and Control of Cancer: Training in Implementation Science; RAND, Research and Development Corporation; ReACH, Research to Advance Community Health; RFA, request for applications; UAMS, University of Arkansas for Medical Sciences; UCI, University of California Irvine; UCLA, University of California Los Angeles; UCR, University of California Riverside; UCSD, University of California San Diego; UCSF, University of California San Francisco; UIC, University of Illinois at Chicago; UNC, University of North Carolina; USC, University of Southern California; UTHSC, The University of Texas Health Science Center; UW, University of Wisconsin; VUMC, Vanderbilt University Medical Center; WUSTL, Washington University in St. Louis.

^a^Please note, given that this data was collected in 2017-2018, it is possible that these CTSA programs have changed or been updated since then.

CTSA leaders sent 1^st^ survey

(n=67)

Table 1 & 2

D&I Experts reporting D&I activity (n=28)

D&I Experts completing 2^nd^ survey (n=35)

D&I Experts sent 2^nd^ survey (n=43)

CTSA leaders completing 1^st^ survey (n=37)

No D&I activity (n=7)

Additional DI&KT WG members surveyed (n=6)

Tables 3 and 4 and Supplementary Material

**Supplementary Figure 1.** *CONSORT Figure.* CTSA, Clinical and Translational Science Award; D&I, dissemination and implementation; DI&KT WG, Clinical and Translational Science Award Dissemination, Implementation, and Knowledge Transfer Working Group.
